# Doses to internal organs for various breast radiation techniques - implications on the risk of secondary cancers and cardiomyopathy

**DOI:** 10.1186/1748-717X-6-5

**Published:** 2011-01-14

**Authors:** Jean-Philippe Pignol, Brian M Keller, Ananth Ravi

**Affiliations:** 1 Radiation Oncology Department, Sunnybrook Health Sciences Centre, Toronto, Ontario, Canada; 2 Medical Physics Departments, Sunnybrook Health Sciences Centre, Toronto, Ontario, Canada

## Abstract

**Background:**

Breast cancers are more frequently diagnosed at an early stage and currently have improved long term outcomes. Late normal tissue complications induced by adjuvant radiotherapy like secondary cancers or cardiomyopathy must now be avoided at all cost. Several new breast radiotherapy techniques have been developed and this work aims at comparing the scatter doses of internal organs for those techniques.

**Methods:**

A CT-scan of a typical early stage left breast cancer patient was used to describe a realistic anthropomorphic phantom in the MCNP Monte Carlo code. Dose tally detectors were placed in breasts, the heart, the ipsilateral lung, and the spleen. Five irradiation techniques were simulated: whole breast radiotherapy 50 Gy in 25 fractions using physical wedge or breast IMRT, 3D-CRT partial breast radiotherapy 38.5 Gy in 10 fractions, HDR brachytherapy delivering 34 Gy in 10 treatments, or Permanent Breast ^103^Pd Seed Implant delivering 90 Gy.

**Results:**

For external beam radiotherapy the wedge compensation technique yielded the largest doses to internal organs like the spleen or the heart, respectively 2,300 mSv and 2.7 Gy. Smaller scatter dose are induced using breast IMRT, respectively 810 mSv and 1.1 Gy, or 3D-CRT partial breast irradiation, respectively 130 mSv and 0.7 Gy. Dose to the lung is also smaller for IMRT and 3D-CRT compared to the wedge technique. For multicatheter HDR brachytherapy a large dose is delivered to the heart, 3.6 Gy, the spleen receives 1,171 mSv and the lung receives 2,471 mSv. These values are 44% higher in case of a balloon catheter. In contrast, breast seeds implant is associated with low dose to most internal organs.

**Conclusions:**

The present data support the use of breast IMRT or virtual wedge technique instead of physical wedges for whole breast radiotherapy. Regarding partial breast irradiation techniques, low energy source brachytherapy and external beam 3D-CRT appear safer than ^192^Ir HDR techniques.

## Background

Breast is the most common site of cancer in women and with the wide-spread use of mammography more than two-thirds of breast cancers are diagnosed at an early stage [1,2]. Early stage breast cancer carries a better prognosis, with outcomes having improved dramatically over the last two decades with a 25% reduction of breast cancer mortality [3]. As patients diagnosed with breast cancer are more likely to survive longer, it is essential to prevent treatment induced fatalities. The main types of radiation therapy induced fatalities that have been widely reported are cardiomyopathy and secondary cancers [4]. Though their occurrence is also influenced by lifestyle and/or a predisposing genetic condition [5,6], it is primarily related to the amount of dose deposited in specific organs [6,7]. So the most efficient way to prevent these sequelae is to reduce the amount of dose scattered to internal organs; for example, choosing a radiation technique that minimizes the exposure of internal organs [5-7]. In regards to secondary cancers, a recent review from Xu *et al. *showed that secondary tumors occur more frequently in organs that are close to radiation fields, in the high/intermediate dose zones [7], and that it is important to assess the scattered dose to those internal organs along with their secondary cancer susceptibility in selecting a radiation technique. In regards to the cardiomyopathy risk, a critical review published by Schultz-Hector stresses the risk of acute dose as low as 1 ~ 2 Gy and a dose-dependent cardiac mortality below 10 Gy [8].

On the other hand, for adjuvant breast radiotherapy, several innovations and new paradigm have been introduced over the last decade. Physical wedge were replaced by virtual wedges, and eventually the dose distribution homogeneity was improved using breast Intensity Modulated Radiation Therapy (IMRT) [9]. Multiple techniques have been proposed for Accelerated Partial Breast Irradiation (APBI) of early stage breast cancer [10,11], which include high dose rate multi-catheter brachytherapy and permanent breast seed implant (PBSI), intra-operative radiotherapy using kilovoltage generator or direct electron beam, and 3D-conformal radiotherapy [12-17].

All of these techniques deliver different levels of scatter doses to internal organs and hence may induce different risks of secondary cancers or cardiomyopathy. The purpose of this paper is to evaluate the amount of scattered dose to internal organs situated in the intermediate/high dose region including the heart, the lung, the contralateral breast and the spleen for different techniques of adjuvant radiotherapy for a typical left sided breast cancer. To avoid confounding factors linked to patient's anatomical characteristics and assess internal organ dose deposition accurately, we used Monte Carlo simulation in an anthropomorphic phantom based on a realistic patient anatomy.

## Methods

### 1 Radiotherapy protocols

Five different breast irradiation protocols were selected: a standard whole breast radiotherapy delivering 50 Gy in 25 treatments to the breast alone, using either physical wedge or virtual wedge/breast IMRT for missing tissue compensation [9,18], partial breast 3D-conformal radiotherapy (3D-CRT) delivering 38.5 Gy in 10 treatments [17], multi-catheter High Dose Rate (HDR) brachytherapy delivering 34 Gy in 10 treatments to the 85% isodose [11,12], and permanent breast seed implants with ^103^Pd seeds delivering a dose of 90 Gy on the Planning Target Volume (PTV) [14].

### 2 Realistic anthropomorphic phantom

A realistic anthropomorphic phantom of a female chest was described in the data entry card of the MCNP Monte Carlo code [19]. This phantom mimicked the planning CT of a small breasted patient randomly selected from the treatment planning database. The geometry modeled was of a typical early stage cancer in the left breast. Complex volumes were build using elementary surfaces combination to create breasts, lungs, heart, chest walls, spleen and other body volumes. Small spherical tally volumes (0.5 to 0.8 cc) were placed in the left and right breasts, on the anterior part of the heart corresponding to the left anterior descending coronary artery [20], and in the posterior part of the ipsilateral lung. A larger spherical tally volume (150 cc) was placed at the position of the spleen, about 5 cm inferiorly to the breast field edge. The MCNP *F8 pulsed height tally function corrected for energy deposition was used to calculate the amount of energy absorbed in each tally volume. This function calculates for each tally the amount of energy deposited minus the energy leaving the volume. Previous work done by our group demonstrated the accuracy of this method in estimating the absorbed dose [21]. These values were converted into dose, accounting for the energy absorbed in the treated breast and the treatment protocol. To facilitate comparison with previously published data, the doses were expressed in Gy (J kg^-1^) when discussing the risk of cardiomyopathy, and in mSv when discussing the risk of secondary cancers.

### 3 External beam radiotherapy

#### 3.1 Hybrid method

Head leakage and room scatter contributions are challenging to assess using Monte Carlo simulation because of the very low probability for a photon to reach a detector inside the phantom. So a hybrid method was used to calculate the scatter dose for external beam radiotherapy techniques. This method adds the dose corresponding to head leakage and room back-scatter measured in a water phantom to the scatter dose produced in beam modifiers and internal phantom scatter calculated using Monte Carlo simulation.

#### 3.2 Head leakage and room back-scatter

The head leakage and room back-scatter contributions were measured in a solid water phantom (Gammax RMI, Middleton, WI) using a Farmer ionization chamber (model 2571). The phantom was placed at a source-axis distance of 100 cm, laterally abutting the central axis of half beam irradiation fields of various sizes: 16 × 20 and 8 × 20 cm^2^. Doses were measured at 5 cm depth in the phantom and at 2.5, 7, 10, 19 and 28 cm away from the beam axis. These scatter doses were interpolated for each field size using a power law.

#### 3.3 Scatter contribution

Dose contributions due to photons scattering from beam modifiers and/or inside the phantom were simulated using the MCNP Monte Carlo code [19]. The photon energy phase-space from a Siemens Primus (Walnut Creek, CA) 6 MV accelerator was pre-calculated [22]. Two opposed parallel beams described as being tangential to the chest wall with a 1 cm lung margin. Field sizes were 16 × 20 cm^2 ^for whole breast irradiation, and 8 × 20 cm^2 ^for the 3D-CRT partial breast irradiation technique. Missing tissue compensation technique used either 30°steel wedges (ρ = 7.81 g.cm^-3^), or field in field segments for about 20% of the dose, the remaining 80% was delivered using open beams. This was done to simulate a virtual wedge/breast IMRT technique.

### 4 Brachytherapy

#### 4.1 Catheter ^192^Ir HDR brachytherapy

A photon energy spectrum with discrete energy probabilities corresponding to ^192^Ir decay was described in the source card. Photons were emitted in 4 π starting randomly from the source placed in the middle of the left breast. The number of photons that were generated was calculated based on the dwell time needed to treat a target volume of 3 cm radius (113 cc), corresponding to a volume of 113 cc, using a 10 Ci source. The Nucletron Plato treatment planning system (Veenendaal, Netherland) was used to calculate the total dwell time, placing catheter evenly spaced every cm across the target volume. In this later case the IPSA dose optimization algorithm was used to generate the dwell positions, to deliver the prescribed dose to the target volume and calculate the total treatment time [23].

#### 4.1 Permanent breast seed implants (PBSI)

The same target volume geometry was used to simulate the PBSI case. A target volume of 113 cc requires a hundred ^103^Pd seeds of 2.7 U, corresponding to a total activity of 0.2088 Ci to deliver a dose of 90 Gy on the minimal peripheral dose [14].

### 5 Risk of secondary cancers estimation

The lifetime probabilities of developing fatal secondary malignancies were calculated per Sv absorbed in breast and lung using the National Council on Radiation Protection and Measurements (NCRP) report 116 Table Seven Part Two page 32 [24].

### 6 Estimation of statistical errors

A typical Monte Carlo result represents the average of the contributions from many particles histories. To calculate this average and the standard deviation the initial problem is divided in several smaller batches. A standard error, *R*, is then calculated as being the ratio between the standard deviation and the average: $R=\frac{S_{\bar{x}}}{\bar{x}}$. A standard error below 5% is generally considered reliable for most calculation. For the current study, the transport of 10^9 ^photons sources was simulated for each opposed beam in order to get reliable estimation of the scattered dose, i.e. with standard error below 1%.

## Results

**Figure 1:**
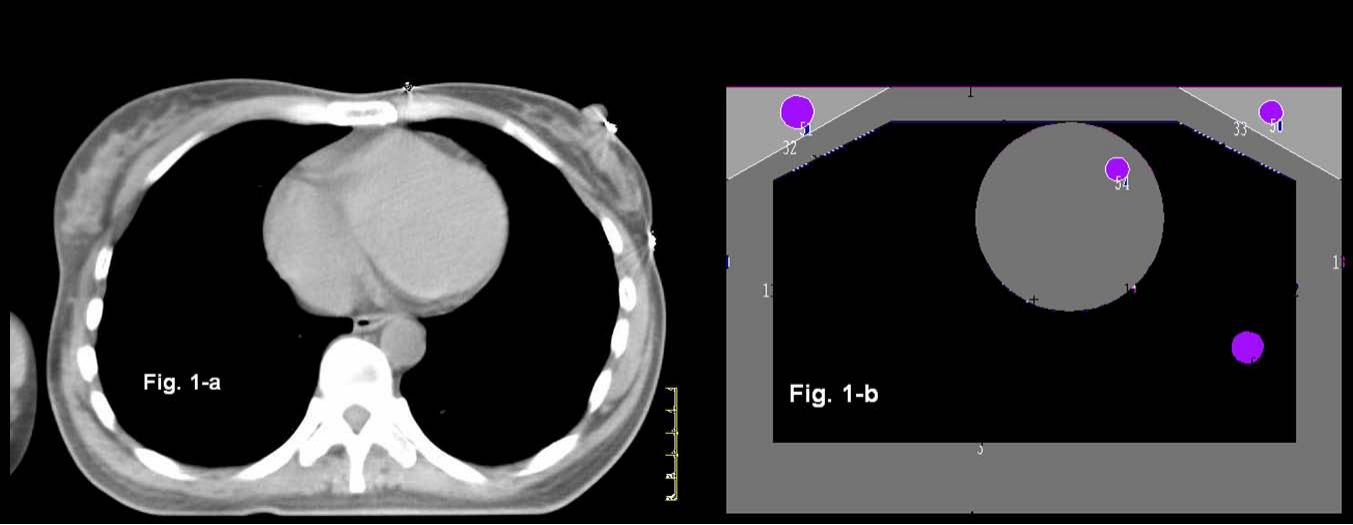
**Planning CT-scan of a typical early stage breast cancer patient with left breast involvement (1-a) and the corresponding volumes described for the Monte Carlo simulation (1-b)**. The pink circles correspond to the Tally detectors placed in the breasts, ipsilateral lung, anterior part of the heart, and the spleen.

Figures 1-a and 1-b show the small breasted patient CT scan and its corresponding phantom designed with MCNP. Overall the phantom was 12 cm height, 26 cm wide and 70 cm long. The breast volumes were 520 cc, corresponding to a typical small/medium breasted patient in a cohort of women treated in a controlled randomized trial in two Canadian institutions [9].

**Figure 2:**
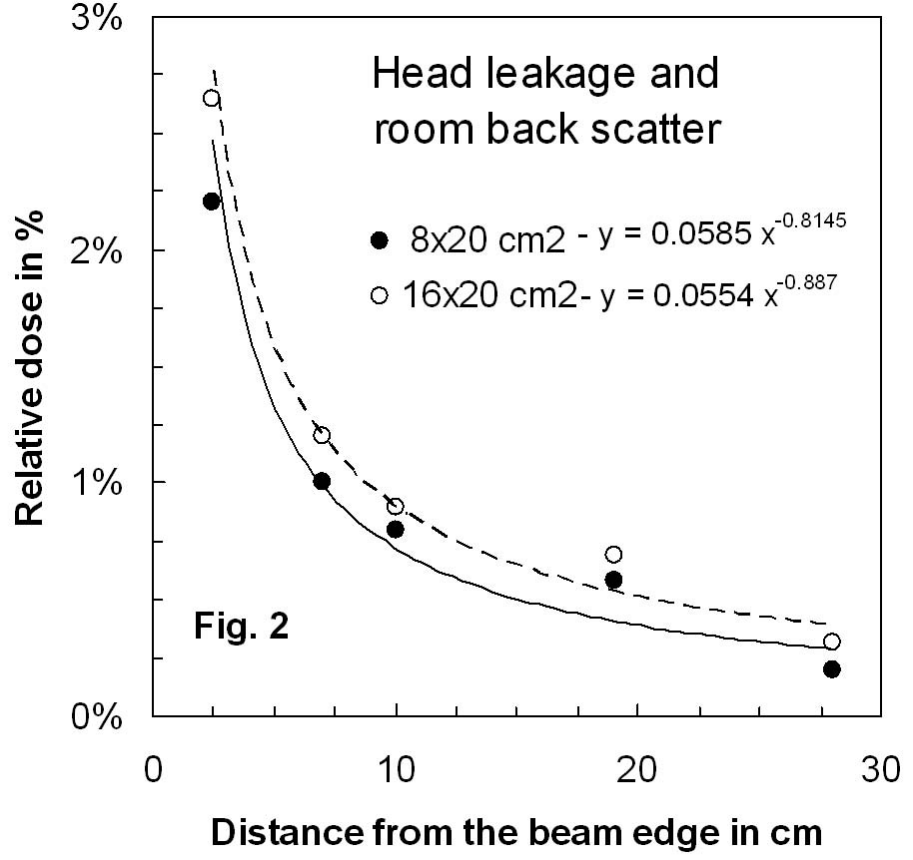
**Relative head leakage and room back scatter contributions measured in a solid water phantom**.

Figure 2 shows the head leakage contribution measured outside the beam boundaries at 5 cm depth in a solid water phantom for the two different field sizes. This contribution is very small, dropping rapidly below 1% of the total dose as the distance from the field edge increase. There is a 20% dose increase for the largest field size that is probably due to the room back-scatter.

Table 1 summarizes the relative contribution to internal organ doses from internal photon scatter, beam modifiers and head leakage for two external beam XRT techniques. The internal scatter is the dominating contribution to the total body dose for breast IMRT while the photon scatter in the wedge compensator accounts for the majority of the scattered dose using physical wedge beam modifiers. Overall, the presence of a physical wedge dramatically increased the dose to most organs outside the treated volume by 50 to 800% compared to breast IMRT.

**Table 1:** Relative contribution from head compensator, leakage and internal scatter to the dose to various organs.

Technique	Breast wedges			Breast IMRT	
	**Internal scatter**	**Compensator**	**Head leakage**	**Internal scatter**	**Head leakage**

Contralateral breast	11.7%	87.8%	0.5%	95.6%	4.4%

Spleen	34.7%	64.7%	0.5%	98.5%	1.5%

Ipsilateral lung	18.7%	79.2%	2.1%	90.1%	9.9%

Heart (anterior 1/3)	38.9%	60.1%	1.0%	97.6%	2.4%

Table 2 compares the dose to selected organs for the various adjuvant breast radiotherapy protocols. There are very large variations of the total body dose between techniques.

**Table 2:** Dose to various organs for various breast radiotherapy techniques.

Technique	PBSI	HDR (catheters)	Wedge	IMRT	3D-CRT
Treated Breast	90 Gy	34 Gy	50 Gy	50 Gy	38.5 Gy

Contralateral Breast	2.2 mSv	230 mSv	1695 mSv	206 mSv	140 mSv

Spleen	44 mSv	1171 mSv	2300 mSv	810 mSv	130 mSv

Ipsilateral lung	790 mSv	2471 mSv	582 mSv	121 mSv	80 mSv

Heart (LAD)	0.7 Gy	3.6 Gy	2.7 Gy	1.1 Gy	0.7 Gy

- For external beam radiotherapy the physical wedge compensation technique yields the largest dose to neighboring solid organs like the spleen or the heart giving respectively 2,356 mSv and 3.0 Gy respectively. Breast IMRT reduces the dose these neighboring organs to 866 mSv and 1.4 Gy respectively, and partial breast irradiation using 3D-CRT is the safest technique with doses of 130 mSv and 0.7 Gy respectively. The dose scattered in the lung is small for IMRT and 3D-CRT, but higher for the wedge technique.

- For partial left breast irradiation using ^192^Ir HDR brachytherapy large doses are scattered to the heart (3.6 Gy), the spleen (1,171 mSv), and the lung (2,471 mSv). Using a balloon catheter these doses are increased by 44% reaching 5.2 Gy to the heart, 1,686 mSv to the spleen and 3,558 mSv to the posterior part of the ipsilateral lung. In contrast, permanent breast seeds implant brachytherapy using low energy source is associated with low doses to most organs despite a higher physical dose is delivered to the target volume. The brachytherapy techniques tend to deliver higher dose to the lung compared to external beam techniques where shielding is used.

## Discussion

This report shows that depending on the radiotherapy techniques large variations, e.g. up to 20 fold for the ipsilateral lung and 800 fold for the contralateral breast, are found in the amount of scattered dose to the organs depending on the adjuvant breast radiation technique. The objective of this work was not to describe the range of scatter doses received by adjuvant breast radiotherapy, since this amount is also highly dependant on other factors including the breast size and side, the location of the surgical cavity for brachytherapy techniques, and the patient body shape and size [5,18]. For example we previously reported up to a 10 fold variation in the dose scattered in the contralateral breast in a prospective study measuring the scatter dose to various body locations in patients receiving standard external beam radiotherapy [18]. To evaluate the long term risks of breast radiotherapy, we compared the scattered dose produced by various radiotherapy techniques while keeping the patient geometry constant. We purposely selected a small left breasted patient to compare the amount of scattered dose for partial breast radiotherapy techniques versus standard whole breast radiotherapy in a worse case scenario.

In regards to secondary cancer, to appreciate the clinical significance of scattered dose one can refer to the critical review published by Eric Hall in 2005 about the increased risk of secondary cancers using conformal IMRT instead of 3D-CRT [5]. In this report, lifetime probabilities of developing fatal secondary malignancies were calculated per Sv absorbed in various organ sites using the National Council on Radiation Protection and Measurements (NCRP) report 116 [24]. Using the same methodology for our study patient, the Table 3 shows the lifetime risk of secondary contralateral breast or lung cancers.

**Table 3:** Lifetime risk of secondary cancers for various breast radiotherapy techniques using the likelihoods from the National Council on Radiation Protection and Measurements (NCRP) Report 116 Table 7.2, page 32.

Cancer type	Probability (%/Sv)	PBSI	HDR (catheters)	Wedge	IMRT	3D-CRT
Breast	0.20	0.00%	0.05%	0.34%	0.04%	0.03%

Lung	0.85	0.67%	2.10%	0.49%	0.10%	0.07%

For the clinical case used in this study the incremental risk of secondary cancer breast cancer is calculated 0.34% for a whole breast technique and wedge compensators. This is likely undetectable compared to the observed frequency of contralateral breast cancer which is about 7% at 10 years and 10% at 15 years [25,26]. For example Obedian did not find significant difference in the occurrence of contralateral breast cancer at 15 years in a retrospective series of 2,416 patients treated with breast conserving surgery and adjuvant radiotherapy or mastectomy without radiotherapy [26]. Though this risk might be higher for younger women or patients with predisposing genetic risks [6,25,27], it remains difficult to detect. Moreover, compared to physical wedge compensation radiotherapy the other techniques, especially the ones delivering partial breast irradiation, yield at least 7 times less scatter dose. So the risk of developing a contralateral breast cancer should be truly undetectable.

For a whole breast technique using physical wedge compensation the lifetime incremental risk of lung cancer is calculated at 0.49%. This value is little higher but of the same order of magnitude than the 0.30% increased risk for adjuvant radiotherapy found by Zablotska on a cohort of 260,000 patients included in the Surveillance Epidemiology and End Results (SEER) database [28]. This difference could be due to the high dose gradient in the lung, the choice of a small breasted women and the position of the detector in the ipsilateral lung that all could increase the amount of scatter dose detected. Nevertheless, from a clinical perspective those rates are small and the risk remains acceptable. Since most radiotherapy techniques except the HDR brachytherapy are yielding similar or lower amount of radiation scatter to the lung they should also be deemed acceptable. The only scenario where a large scatter dose is found in the lung is ^192^Ir HDR brachytherapy. This is likely due to the limited absorption in the lung tissue of the high energy photons (average energy 367 keV) that are emitted in all directions and without shielding from the ^192^Ir source. The risk of secondary lung cancer calculated in this case is increased by a factor 4, with 2 in 100 women at risk of developing lung cancer.

Regarding cardiac risk, a recent critical review published by Schultz-Hector suggest that acute single dose of 1~2 Gy to the heart increased the risk of developing ischemic heart disease significantly [8]. And the excess relative risk could be linearly fitted with a slope of 17% per Gy. Bearing in mind those values, external beam radiotherapy with physical wedge compensation and HDR breast brachytherapy which yield excess dose to the heart are deemed inappropriate breast adjuvant radiotherapy techniques. Since the use of ^103^Pd has a strong protecting effect on the heart dose, the low energy photons being absorbed rapidly in the tissue, alternative sources like low energy electronic or ^169^Yb sources should be considered for HDR applications [29,30].

## Conclusions

Since the majority of women eligible for breast conserving therapy have improved outcomes, they are likely to live long enough to develop secondary cancers or cardiac failures and it is important to prevent those morbidities when considering a new technique. Whole breast radiotherapy, breast IMRT and virtual wedges appears safer than physical wedge compensation, and for partial breast irradiation techniques, external beam 3D-CRT and low energy source brachytherapy appear safer than ^192^Ir HDR techniques.

## Competing interests

The authors declare that they have no competing interests.

## Authors' contributions

JPP realized the Monte Carlo simulation, analyzed the data and wrote the manuscript. He is the corresponding author. BMK reviewed the Monte Carlo simulation and the data analysis. He realized the experimental water phantom measurements of the head leakage and room back-scatter. He carefully reviewed the manuscript. AR did the planning of the brachytherapy treatments, checked all the calculations and carefully reviewed the manuscript. All the authors read and approved the final manuscript.
